# A phase II study of afatinib, an irreversible ErbB family blocker, added to letrozole in patients with estrogen receptor-positive hormone-refractory metastatic breast cancer progressing on letrozole

**DOI:** 10.1186/s40064-015-1601-7

**Published:** 2016-01-19

**Authors:** Katharina Gunzer, Florence Joly, Jean-Marc Ferrero, Joseph Gligorov, Helene de Mont-Serrat, Martina Uttenreuther-Fischer, Katy Pelling, Sven Wind, Guilhem Bousquet, Jean-Louis Misset

**Affiliations:** Oncologue-Service de Recherche Clinique, Centre François Baclesse, Avenue Général Harris, 14076 Caen Cedex 05, France; Centre Hospitalier Universitaire Côte de Nacre, Caen, France; Centre Antoine Lacassagne, Nice, France; APHP, HUEP-Tenon Hospital, Paris, France; Institut Universitaire de Cancérologie, UPMC, Paris, France; Boehringer Ingelheim France S.A.S., Reims, France; Boehringer Ingelheim Pharma GmbH and Co. KG, Biberach, Germany; Boehringer Ingelheim Ltd., Bracknell, UK; Hôpital Saint Louis, Paris, France

**Keywords:** Phase II, Afatinib, ErbB Family Blocker, Letrozole, Metastatic breast cancer, Pharmacokinetics

## Abstract

Phase II, open-label study assessing the efficacy and safety of the ErbB family blocker afatinib combined with letrozole in estrogen receptor-positive metastatic breast cancer (MBC) patients who had progressed on letrozole monotherapy. Adult females (*N* = 28) received oral afatinib (50 [*n* = 7], 40 [*n* = 13] or 30 [*n* = 8] mg/day) plus letrozole 2.5 mg/day in 28-day cycles until disease progression. Primary endpoint was the progression-free rate at or after 16 weeks of afatinib. At 16 weeks, four patients remained on afatinib without progression; two of these were HER2 negative. Fifteen (54 %) patients had a best response of stable disease according to Response Evaluation Criteria in Solid Tumors. Median progression-free survival was 60, 107 and 79 days with 50, 40 and 30 mg/day afatinib, respectively. Diarrhea, asthenia, rash, mucosal inflammation and nausea were the most frequent adverse events. In this small, exploratory study, afatinib combined with letrozole was able to induce disease stabilization in 54 % of hormone-refractory MBC patients previously progressing on letrozole.

Clinical trial registration: NCT00708214

## Background

Abnormally activated ErbB Family receptors (EGFR [ErbB1], HER2 [ErbB2], ErbB3 and ErbB4) and estrogen receptors (ER) are frequently implicated in breast cancer, making these potential therapeutic targets (Hurvitz and Pietras 2008; Ciardiello and Tortora 2008). Specifically, anti-estrogens, aromatase inhibitors (AIs) and selective ER modulators have proven effective in the treatment of hormone receptor-positive breast cancer; however, a major clinical problem limiting the usefulness of endocrine therapy is tumor resistance (Schiff et al. 2004; Chu and Lu 2008).

Exploration into the complexity of receptor signaling has identified that receptor crosstalk constitutes a fundamental mechanism of tumor resistance, and preclinical observations have confirmed that activation of ER and ErbB Family receptors, namely EGFR/HER2, on the tumor cell surface stimulates multiple intracellular signaling cascades, leading to cellular proliferation, survival and differentiation (Ullrich and Schlessinger 1990; Klapper et al. 2000). Bidirectional crosstalk can lead to cross-activation of these downstream signaling pathways, resulting in the development of tumor resistance to endocrine therapy (Nicholson et al. 2001; Arpino et al. 2008; Hurtado et al. 2008). To overcome such resistance, the combination of an AI with a tyrosine kinase inhibitor (TKI) targeting either EGFR or HER2 has been explored and has been shown to enhance response and progression-free survival (PFS) compared with treatment with an AI alone (Johnston et al. 2009; Cristofanilli et al. 2010). Such findings confirm that combining endocrine treatment with an ErbB Family-targeted therapy could be an effective strategy in the management of patients with primary or acquired hormone-resistant breast cancer (Osborne et al. 2005). Furthermore, restoration of hormone sensitivity via ErbB Family inhibition could pose a therapeutic advantage, providing sustained disease control without the need for chemotherapy.

Afatinib (BIBW 2992) is an orally available, irreversible ErbB Family Blocker, with a half maximal inhibitory concentration of 0.5 nM, 14 and 1 nM for EGFR, HER2 and ErbB4, respectively (Li et al. 2008). This compound also inhibits ErbB3 transphosphorylation (Li et al. 2008). Phase I monotherapy studies in patients with advanced solid tumors recommended a continuous dose of afatinib 50 mg once daily for the phase II setting (Yap et al. 2010; Agus et al. 2006). Afatinib monotherapy (50 mg/day) has previously demonstrated clinical activity in heavily pre-treated patients with HER2-positive metastatic breast cancer (MBC) who had progressed following trastuzumab treatment, with a median PFS of 15 weeks and an overall survival (OS) of 61 weeks (Lin et al. 2012), as well as a small proportion of patients with triple-negative MBC who had progressed following no more than two prior lines of chemotherapy (Schuler et al. 2010). To further investigate the potential activity of afatinib in MBC, we undertook a phase II exploratory study to assess the efficacy, safety and pharmacokinetics of afatinib in combination with letrozole in patients with ER-positive MBC who had progressed on letrozole monotherapy.

## Patients and methods

### Study design

This was a phase II, open-label, single-arm, multicenter trial of afatinib added to letrozole in patients with ER-positive MBC who had progressed on letrozole monotherapy. Patients continued to receive letrozole monotherapy during the 2-week screening period. The primary efficacy endpoint was progression-free rate at or after 16 weeks of afatinib treatment. Progression was defined as the occurrence of any of the following: new bone lesion(s) identified on a bone scan or with magnetic resonance imaging (MRI), progression or occurrence of new lesion(s) according to the Response Evaluation Criteria In Solid Tumors (RECIST) version 1.0 (Therasse et al. 2000), an increase in the tumor marker CA 15.3 of more than 20 % compared with the baseline value (at two consecutive examinations), or disease-related skeletal events (pathological or vertebral compression fracture not related to trauma; palliative radiotherapy for bone pain; prophylactic radiation or surgery for an impending fracture; spinal cord compression). Secondary endpoints included objective response (complete response [CR] or partial response [PR]) based on RECIST, clinical benefit (CR or PR or stable disease [SD]) according to RECIST at 16 and 24 weeks, PFS, OS, time to and duration of objective response, pharmacokinetics and safety.

The study was conducted between 30 May 2007 and 4 January 2010 at five sites in France, and was in accordance with the Declaration of Helsinki, the International Conference on Harmonization Good Clinical Practice Guideline and approved by the French national regulatory agency and local ethics committees. All patients provided written informed consent prior to study participation.

### Study population

Eligible females (≥18 years) had histologically confirmed stage IIIB or IV ER-positive MBC. Patients had previously received letrozole and had developed acquired resistance, defined as disease progression on letrozole following previous response (PR or better, or SD for ≥24 weeks). Initially, only patients who had not received prior chemotherapy for metastatic disease were allowed to participate. Following an amendment, up to two lines of prior chemotherapy for MBC were permitted. Patients with HER2-positive breast cancer could have received prior trastuzumab in the adjuvant and metastatic setting. HER2 positivity was not a requirement for study entry. Further eligibility criteria included: an Eastern Cooperative Oncology Group performance status of 0 or 1; life expectancy of ≥6 months; recovery from any grade 3 (according to National Cancer Institute Common Terminology Criteria for Adverse Events [NCI CTCAE] version 3.0) adverse events (AEs) from previous treatment; documented menopause (estradiol level of <11 pg/mL); diagnosis of disease progression ≤6 weeks prior to trial entry; and adequate bone marrow (absolute neutrophil count ≥1500/mm^3^, platelet count ≥100,000/mm^3^), hepatic (aspartate aminotransferase and/or alanine aminotransferase ≤3× the upper limit of normal) and renal (serum creatinine ≤1.5 mg/dL [≤132 μmol/L, SI unit or equivalent]) function. Patients were excluded from the study if they had: chronic diarrhea or gastrointestinal (GI) disorders that could interfere with absorption of study treatment; brain metastases; significant cardiovascular disease (i.e., uncontrolled hypertension, unstable angina pectoris, history of infarction, congestive heart failure of New York Heart Association grade >2) within the past 12 months; received chemotherapy, immunotherapy, radiotherapy, other EGFR- and/or HER2-inhibiting drugs, or had participated in another clinical study within 4 weeks before the start of therapy. Treatment with bisphosphonates was allowed if the patient had been receiving a stable dose for at least 3 months prior to study entry.

### Treatment

Patients received oral afatinib once daily at a starting dose of 50 mg (which was reduced to 40 mg and then to 30 mg following protocol amendments) over repeated 28-day treatment cycles, with 2.5 mg of oral letrozole daily until disease progression, withdrawal of consent or discontinuation due to AEs. The study medications were administered concurrently 1 h prior to food intake. The protocol amendments were made as some patients receiving afatinib 50 or 40 mg experienced grade 3 cutaneous AEs and diarrhea. As a result, the starting dose of afatinib was reduced to 40 mg once-daily and then 30 mg once-daily for all newly enrolled patients. At each starting dose, dose reduction of afatinib in 10 mg decrements (to a minimum of 20 mg) was a pre-specified option for patients experiencing certain drug-related AEs. A safety analysis was performed when the initial 15 patients had completed 1 month of treatment at either 50 or 40 mg afatinib. This analysis was supportive of the reduction of the afatinib starting dose from 40 to 30 mg per day. Concomitant treatments were permitted as clinically necessary.

### Efficacy assessments

Efficacy was assessed in terms of the progression-free rate at or after 16 weeks of treatment. If a patient withdrew from the study due to clinical deterioration, according to the investigator’s judgment, this was considered as progressive disease (PD). Target lesions were evaluated by clinical detection, computed tomography or MRI at screening and at the end of every other 28-day treatment cycle.

### Safety assessments

Safety was assessed by monitoring the incidence and intensity of AEs that patients experienced. AEs were graded according to the NCI CTCAE version 3.0.

### Pharmacokinetic sampling and data analysis

For quantification of drug plasma concentrations, 5 mL of venous blood was collected. Blood samples were drawn before and 2 h after letrozole administration on day –14 and –7 during the first 2 weeks of letrozole monotherapy treatment. For quantification of letrozole and afatinib plasma concentrations, blood samples were drawn on day 1 (cycles 1, 3, 4 and 5) before and 2 h after administration of afatinib and letrozole, and additionally at 1, 2, 4, 6, 8 and 24 h after drug administration on day 1 of cycle 4. Afatinib plasma concentrations were determined by a validated high performance liquid chromatography-mass spectrometry (HPLC–MS/MS) assay at the Department of Drug Metabolism and Pharmacokinetics, Boehringer Ingelheim Pharma GmbH and Co. KG, Biberach, Germany. Letrozole plasma concentrations were determined by a validated HPLC–MS/MS assay at Nuvisan Pharma Services GmbH & Co. KG, Neu-Ulm, Germany.

### Statistical analyses

The analyses in this trial were descriptive and exploratory. A sample size of 30–40 patients was required to provide 80 % power to detect statistically significant evidence of afatinib activity, based on the assumption of an underlying true rate of non-progression of 25 % at 16 weeks. Sample size was based on a one-sided alpha of 10 %. Analysis was completed on the treated set, comprising all patients who received at least one dose of afatinib.

## Results

### Patient population

**Fig. 1:**
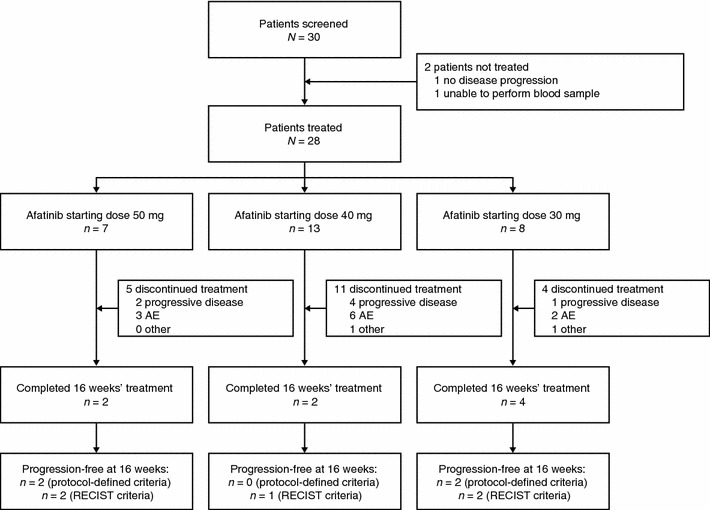
Trial patient disposition

Of a total of 30 patients screened, 28 entered the trial and received at least one dose of afatinib (Fig. 1). Of the two patients who did not enter the trial, one patient did not have disease progression and for the other, it was not possible to perform blood samples. Baseline patient characteristics are shown in Table 1. Most patients (18; 64 %) had experienced SD ≥24 weeks as their best response to prior letrozole therapy, with six patients (21 %) experiencing a PR, three patients (11 %) experiencing a CR and one patient (4 %) having SD (16 weeks).

**Table 1:** Demographics and characteristics of treated patients

	*N* (%)^a^
Total number	28 (100)
Sex	
Female	28 (100)
Age (years)	
Mean	62.5
Median	64
Range	40–82
Baseline ECOG performance status	
0	16 (57)
1	8 (29)
2^b^	4 (14)
Progesterone receptor positive	20 (71)
Estrogen receptor positive	27 (96)
HER2 status	
HER2 positive	3 (11)
HER2 negative	20 (71)
HER2 unknown or missing	5 (18)
Prior therapies	
Chemotherapy	24 (86)
Prior adjuvant or neoadjuvant chemotherapy	
Yes	19 (68)
No	9 (32)
Number of prior metastatic chemotherapies	
0	18 (64)
1	9 (32)
≥2	1 (4)
Hormone therapy prior to letrozole	28 (100)
Prior adjuvant radiotherapy	
Yes	16 (57)
No	12 (43)
Number of prior metastatic radiotherapies	
0	16 (57)
1	7 (25)
≥2	7 (18)

*ECOG* Eastern Cooperative Oncology Group, *HER2* human epidermal growth factor receptor 2

^a^Unless otherwise specified

^b^At screening, the ECOG PS for these patients was 0–1; however, their scores worsened to 2 prior to the start of treatment

### Clinical activity

#### Progression-free rate at 16 weeks (primary endpoint)

A total of 28 patients were evaluable for tumor response. Twenty patients (71 %) discontinued treatment prior to the 16-week endpoint and eight patients completed 16 weeks of treatment (Fig. 1). Of these, four (14 %) were progression free according to the criteria defined for the primary endpoint (Table 2). Two of these patients were HER2 negative and the HER2 status was unknown for the other two.

**Table 2:** Summary of patients reaching the primary endpoint (progression-free rate at 16 weeks)

	Afatinib 50 mg + letrozole 2.5 mg	Afatinib 40 mg + letrozole 2.5 mg	Afatinib 30 mg + letrozole 2.5 mg	All afatinib doses
Number of patients, *n* (%)	7 (100.0)	13 (100.0)	8 (100.0)	28 (100.0)
Progression-free rate (Protocol-defined criteria)^a^, *n* (%) [95 % CI]	2 (28.57) [3.67–70.96]	0 (0.00) [0.00–24.71]	2 (25.00) [3.19–65.09]	4 (14.29) [4.03–32.67]
Progression-free rate (RECIST criteria), *n* (%) [95 % CI]	2 (28.57) [3.67–70.96]	1 (7.69) [0.19–36.03]	2 (25.00) [3.19–65.09]	5 (17.86) [6.06–36.89]

*CI* confidence interval, *MRI* magnetic resonance imaging, *RECIST* Response Evaluation Criteria In Solid Tumors

^a^Progression was defined as the occurrence of one of the following: new bone lesion(s) on bone scan or on MRI; progression or occurrence of new lesion(s) according to RECIST; an increase in the tumor marker CA 15.3 of more than 20 % compared with the baseline value (at two consecutive examinations); or disease-related skeletal events

Of the four who completed 16 weeks of treatment but were not progression-free according to the primary endpoint definition, two were diagnosed with PD at the 16-week assessment. One patient, who received 40 mg afatinib, was considered progression-free according to RECIST criteria, but had elevated CA 15.3 levels. The final patient had suspected RECIST progression prior to 16 weeks which was not formally documented due to difficulty of determining on computed tomography.

**Fig. 2:**
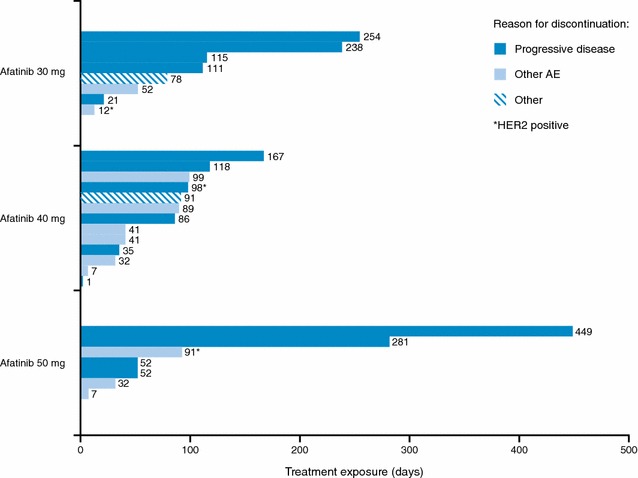
Duration of therapy by starting dose of afatinib for patients who discontinued treatment (each *bar* represents one patient)

For those who discontinued the trial due to PD (n = 15; Fig. 2), 13 had RECIST progression, one patient had elevated CA 15.3 levels and bone lesion progression and one patient had other criteria indicating PD.

### Objective response and clinical benefit rate (RECIST)

No patients experienced an objective (complete or partial) tumor response to afatinib plus letrozole treatment. Throughout the study, a best response of SD, as determined by RECIST, was experienced by 15 patients (54 %). The incidence of SD was comparable across the afatinib starting dose cohorts. Overall, six patients (21 %) experienced clinical benefit at 16 weeks; this included the four patients who met the primary endpoint, the patient who was progression-free based on RECIST (but had elevated CA 15.3 levels) and one patient who had SD at week 16 but had discontinued treatment. Of these six patients, the best response on prior letrozole therapy was SD for five and PR for one patient. Four (14 %) of these six patients continued to have clinical benefit at 24 weeks. Best response to previous letrozole treatment was PR in one patient and SD in three patients.

### Progression-free survival and overall survival

According to RECIST criteria, median PFS (25th percentile, 75th percentile) was 60 (51, 274), 107 (65, 116) and 79 (51, 230) days for patients initially receiving 50, 40 and 30 mg afatinib, respectively. The longest duration of treatment with afatinib and letrozole was 449 days. Duration of therapy, along with reasons for discontinuation, is shown by starting dose of afatinib for all patients (Fig. 2). Median OS could not be estimated due to the small number of patients who died during the trial (three patients died during the trial; none of the deaths were considered treatment-related).

### Safety

All 28 patients experienced AEs considered to be treatment related by the investigator (Table 3). The most frequently reported treatment-related AEs were diarrhea (26 patients; 93 %), asthenia and rash (both 16 patients; 57 %), mucosal inflammation (11 patients; 39 %) and nausea (10 patients; 36 %). The most frequently occurring grade ≥3 treatment-related AEs were diarrhea (six patients; 22 %), asthenia and rash (five patients each; 18 %) (Table 3). No treatment-related grade 5 AEs occurred, and only one patient who received afatinib 40 mg and letrozole 2.5 mg experienced a grade 4 AE (diarrhea). Fourteen patients (50 %) discontinued trial treatment due to treatment-emergent AEs. The most frequent AEs necessitating treatment discontinuation were diarrhea (eight patients; 29 %), asthenia (four patients; 14 %), rash and mucosal inflammation (three patients each; 11 %); some patients had more than one AE leading to treatment discontinuation.

**Table 3:** Treatment-related adverse events by afatinib starting dose and reported as NCI CTCAE grades (patients with at least 1 grade ≥3 adverse event; treated set)

	Afatinib 50 mg + letrozole 2.5 mg, *n* = 7		Afatinib 40 mg + letrozole 2.5 mg, *n* = 13		Afatinib 30 mg + letrozole 2.5 mg, *n* = 8		All afatinib doses + letrozole 2.5 mg, *n* = 28	
	*n* (%)		*n* (%)		*n* (%)		*n* (%)	
	All grades	Grade ≥ 3	All grades	Grade ≥ 3	All grades	Grade ≥ 3	All grades	Grade ≥ 3
Patients with any treatment-related adverse event	7 (100.0)	7 (100.0)	13 (100.0)	8 (61.5)	8 (100.0)	4 (50.0)	28 (100.0)	19 (68)
Adverse event								
Diarrhea	7 (100.0)	1 (14.3)	12 (92.3)	4 (30.8)	7 (87.5)	1 (12.5)	26 (92.9)	6 (21.5)
Rash	5 (71.4)	5 (71.4)	5 (38.5)	0	6 (75.0)	0	16 (57.1)	5 (17.9)
Asthenia	4 (57.1)	2 (28.6)	7 (53.8)	1 (7.7)	5 (62.5)	2 (25.0)	16 (57.1)	5 (17.9)
Mucosal inflammation	5 (71.4)	2 (28.6)	4 (30.8)	2 (15.4)	2 (25.0)	0	11 (39.3)	4 (14.3)
Nausea	3 (42.9)	0	4 (30.8)	0	3 (37.5)	1 (12.5)	10 (35.7)	1 (3.6)
Acne	5 (71.4)	3 (42.9)	3 (23.1)	1 (7.7)	0	0	8 (28.6)	4 (14.3)
Dermatitis acneiform	0	0	5 (38.5)	1 (7.7)	1 (12.5)	0	6 (21.4)	1 (3.6)
Vomiting	1 (14.3)	0	3 (23.1)	1 (7.7)	1 (12.5)	1 (12.5)	5 (17.9)	2 (7.1)
Paronychia	1 (14.3)	0	1 (7.7)	1 (7.7)	2 (25.0)	0	4 (14.3)	1 (3.6)
Dehydration	0	0	1 (7.7)	1 (7.7)	1 (12.5)	0	2 (7.1)	1 (3.6)
Cheilitis	1 (14.3)	0	1 (7.7)	1 (7.7)	0	0	2 (7.1)	1 (3.6)
Skin toxicity	0	0	1 (7.7)	1 (7.7)	0	0	1 (3.6)	1 (3.6)
Arthritis bacterial	0	0	1 (7.7)	1 (7.7)	0	0	1 (3.6)	1 (3.6)
Pneumococcal sepsis	0	0	1 (7.7)	1 (7.7)	0	0	1 (3.6)	1 (3.6)
Renal failure acute	0	0	1 (7.7)	1 (7.7)	0	0	1 (3.6)	1 (3.6)

*NCI CTCAE* National Cancer Institute Common Terminology Criteria for Adverse Events (version 3.0)

Six of the seven patients (86 %) who initially received 50 mg afatinib underwent protocol-specified dose reduction to 40 mg for a grade 3 AE. For two of these six patients the dose was subsequently further reduced to 30 mg because they experienced another grade 3 AE. Eight of 13 patients (62 %) who initially received 40 mg afatinib underwent dose reduction to 30 mg. None of the eight patients who received the 30 mg afatinib starting dose required dose reduction.

The frequency of AEs observed varied across different doses of afatinib. A higher than expected rate of grade 3 GI and skin-related AEs (rash, acne, dermatitis acneiform and palmar–plantar erythrodysesthesia) was observed at the 50 mg starting dose. However, patients fully recovered with treatment interruption or discontinuation. Further analyses suggested dose-related trends for both rash and diarrhea. Among the 16 patients (57 %) who experienced treatment-related rash, those receiving higher doses of afatinib were more likely to experience grade 3 rash and have a faster onset of symptoms (7 days from the start of therapy for patients receiving 50 mg or 40 mg compared with 8–14 days for patients receiving 30 mg). There was a small decrease in the incidence of drug-related diarrhea with decreasing dose. The first onset of treatment-related diarrhea usually occurred within 7 days after the start of therapy.

### Pharmacokinetics

#### Afatinib

Table 4 shows the geometric mean pharmacokinetic parameters of afatinib after multiple administrations of afatinib 30 mg or 40 mg in combination with letrozole 2.5 mg. Inter-subject variability for maximum plasma concentration at steady state (C_max,ss_) and area under the plasma concentration–time curve at steady state over the dosing interval (AUC_tau,ss_) was moderate for the afatinib 40 mg dose group and high for the 30 mg dose group. In general, the pharmacokinetic data available were sparse, and for the 50 mg afatinib dose group there were insufficient data to perform any descriptive statistics.

**Table 4:** Summary of afatinib pharmacokinetic parameters after multiple oral administrations of afatinib 40 and 30 mg, with letrozole 2.5 mg

Pharmacokinetic parameter (unit)	Afatinib 40 mg			Afatinib 30 mg		
	*N*	Geometric mean	Geometric CV (%)	*N*	Geometric mean	Geometric CV (%)
AUC_tau,ss_ (ng*h/mL)	4	660	41.3	5	579	62.8
C_max,ss_ (ng/mL)	4	43.8	42.0	5	33.9	79.4
C_pre,ss, 57_ (ng/mL)	5	21.4	42.3	8	15.8	42.1
C_pre,ss, 85_ (ng/mL)	4	16.9	55.7	5	17.3	46.5
t_max,ss_ (h)^a^	4	2	2–4	5	4.00	0.91–7.67

*AUC*_*tau,ss*_ area under the plasma concentration–time curve at steady state over the dosing interval, *C*_*max,ss*_ maximum plasma concentration at steady state, *C*_*pre,ss, 57*_ *, C*_*pre,ss, 85*_ pre-dose plasma concentration on day 1 of cycles 3 and 4, respectively, *CV* coefficient of variation, *t*_*max,ss*_ time to maximum plasma concentration at steady state

^a^Median and range

#### Letrozole

**Fig. 3:**
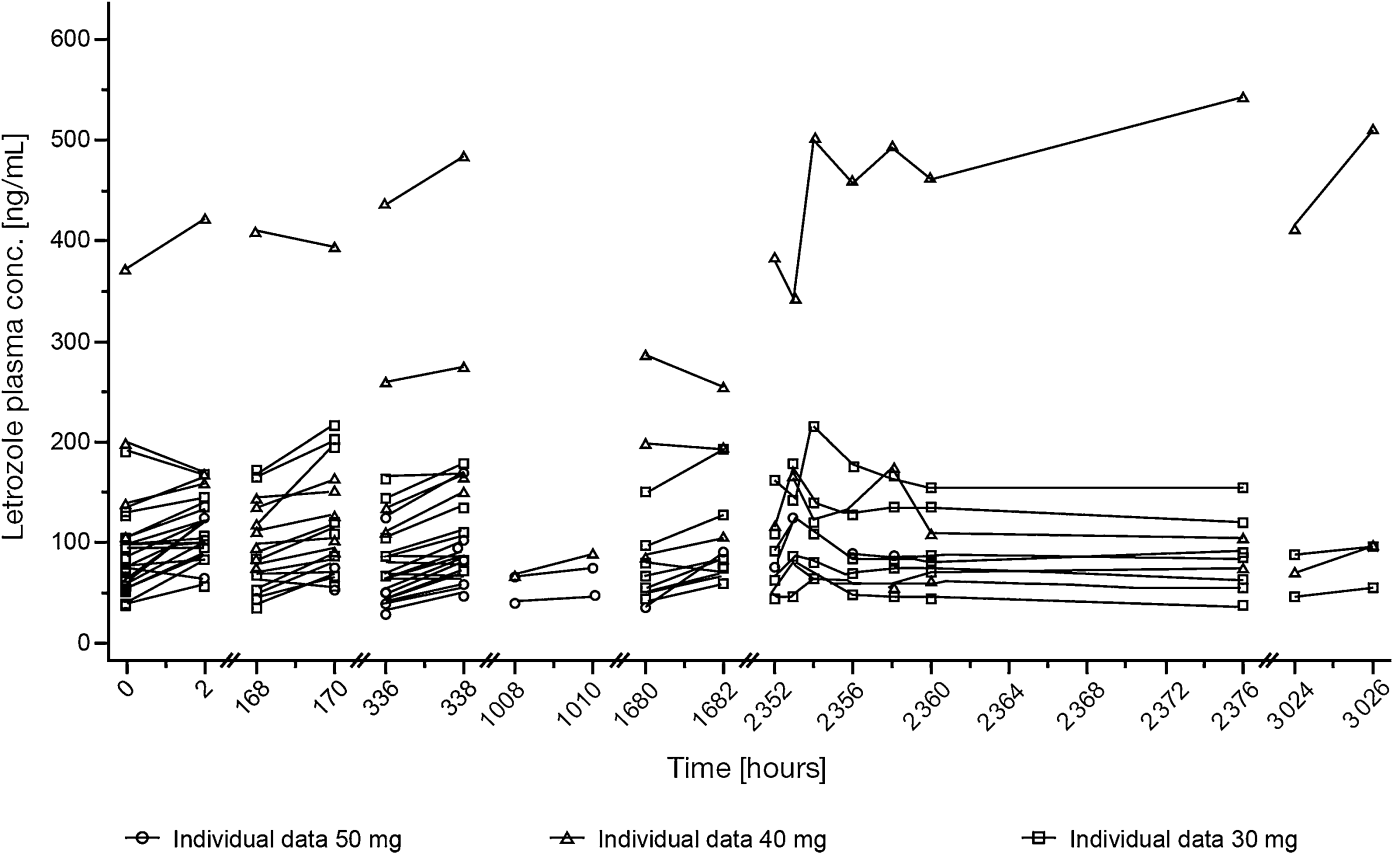
Individual plasma concentration–time profiles of letrozole after multiple oral administrations of 30, 40 or 50 mg afatinib

Letrozole trough and 2-h post-dose plasma concentrations were comparable for the screening period (without afatinib administration) and the afatinib treatment period, indicating that there was no effect of afatinib on letrozole exposure (Fig. 3). In line with this, letrozole plasma concentrations appeared to be independent of the afatinib dose level. Letrozole pharmacokinetic parameters on day 1 of cycle 4 were summarized over all afatinib dose groups (geometric mean C_max,ss_ was 135 ng/mL and geometric mean AUC_tau,ss_ was 2420 ng*h/mL). The inter-subject variability for the overall pharmacokinetic parameters was high.

## Discussion

This phase II study evaluated daily afatinib (30, 40 or 50 mg/day) in combination with letrozole in ER-positive, hormone-refractory MBC. The attempt to overcome hormone resistance, i.e. resistance to letrozole by the addition of the ErbB Family inhibitor afatinib, achieved disease stabilization in four patients (14 %) who remained on treatment without progression after 16 weeks. Two of these patients were HER2-negative. The use of the additional criteria for the diagnosis of progression for the primary endpoint was felt important at the time of this study as bone lesions were deemed non-measurable by RECIST version 1.0. Patients with ER-positive breast cancer frequently have bone metastases. Upon PD during trial treatment, chemotherapy was available to patients as an additional option. In order not to withhold chemotherapy from these patients, close monitoring of PD in bone lesions, which was not necessarily captured by RECIST 1.0, was required. Overall, the ability of afatinib, when administered with letrozole, to induce SD in around 50 % of patients may indicate the potential for reversal of hormone resistance with this agent. Of note, the duration of endocrine therapy prior to afatinib treatment in those who did and did not achieve clinical benefit was not available for all patients, so we are unable to speculate as to whether indolent disease contributed to any treatment effect in this study.

Clinical evidence that crosstalk between the ER and ErbB Family receptors constitutes a mechanism for resistance to endocrine therapy is continually growing. The membrane-bound ER can directly interact with and/or activate a variety of receptors, including EGFR and HER2. HER2 does not have a specific ligand but is able to dimerize with EGFR or ErbB3 as a result of its open conformation (Schiff et al. 2004). Therapeutic strategies focused around combining endocrine therapy with ErbB Family inhibitors, to circumvent de novo or acquired resistance, are being explored. Phase II trials investigating gefitinib or erlotinib, two reversible EGFR TKIs, in combination with an AI in ER-positive breast cancer patients have demonstrated varying levels of clinical benefit, including prolonging PFS, using this combined approach (Table 5) (Cristofanilli et al. 2010; Mayer et al. 2006; Osborne et al. 2011; Smith et al. 2007; Mita et al. 2005). In our phase II trial, among 28 patients evaluable for response, six (21 %) experienced clinical benefit at 16 weeks and median PFS was 60, 107 and 79 days for patients receiving 50, 40 and 30 mg afatinib, respectively, demonstrating that the combination of afatinib and letrozole may warrant further investigation for hormone-refractory MBC patients.

**Table 5:** Clinical trials co-targeting growth factor receptors in hormone-positive metastatic breast cancer patients

Study/phase	Patients	Prior hormone therapy experience	*N*	Regimen	Median PFS (months)	Grade ≥3 AEs
EGFR inhibitors						
Phase II (Mayer et al. 2006)	ER/PgR-positive MBC	Hormone-sensitive population	150	Letrozole + erlotinib	Of 22 patients enrolled, 11/20 patients achieved clinical benefit (1 CR, 4 PR and 6 SD)	Hypermagnesemia, hypokalemia and scalp infection in 1 patient
Phase II (Mita et al. 2005)	ER-positive MBC	Hormone-refractory population	15	Anastrozole + gefitinib	No clinical benefit	–
Phase II (Cristofanilli et al. 2010)	ER/PgR-positive MBC	No prior endocrine therapy and/or developed metastatic disease during/after adjuvant tamoxifen	93^a^	Anastrozole + gefitinib	14.7	Diarrhea 7 vs. 0 % Fatigue 5 vs. 4 % Hot flash 2 vs. 0 %
				Anastrozole + placebo	8.4	
Phase II (Osborne et al. 2011)	ER/PgR-positive MBC	Patients with newly metastatic disease or recurring after adjuvant tamoxifen	206^b^	Tamoxifen + gefitinib	10.9	Diarrhea 4 vs. 0 % Rash 4 vs. 0 % Vomiting 2 vs. 2 %
				Tamoxifen + placebo	8.8	
	ER/PgR/HER2-positive MBC		37	Tamoxifen + gefitinib	6.7	–
				Tamoxifen + placebo	5.8	
TAnDEM/phase III (Kaufman et al. 2009)	ER/PgR/HER2-positive MBC	Previous treatment with tamoxifen as adjuvant or hormonal therapy for MBC or anastrozole was permitted (begun up to 4 weeks before random assignment)	207	Anastrozole + trastuzumab	4.8 (independent assessment)	Vomiting 3 vs. 1 % Hypertension 2 vs. 4 %
				Anastrozole	2.4 (independent assessment)	
EGF30008/phase III(Johnston et al. 2009)	ER/PgR/HER2-positive MBC^c^	No prior therapy for advanced or metastatic disease was allowed. Prior neoadjuvant/adjuvant antiestrogen/adjuvant aromatase inhibitor and/or trastuzumab, (completed >1 year before study entry)	219	Letrozole + lapatinib	8.2	Diarrhea 10 vs. <1 % Rash 1 vs. 0 %
				Letrozole + placebo	3.0	
	ER/PgR-positive HER2-negative MBC (relapsed <6 months after stopping adjuvant tamoxifen)		200	Letrozole + lapatinib	8.3	–
				Letrozole + placebo	3.1	
	ER/PgR-positive HER2-negative MBC with low ER expression (ER H-score <160) (Finn et al. 2009)		207	Letrozole + lapatinib	13.6	
				Letrozole + placebo	6.6	
eLEcTRA/phase III(Huober et al. 2012)	ER/PgR/HER2-positive MBC	First-line treatment; no prior hormone therapy	26	Letrozole + trastuzumab	14.1	Bone pain 4 vs. 7 % Dyspnea 0 vs. 7 %
			31	Letrozole	3.3	
	ER/PgR-positive HER2-negative MBC		35	Letrozole	15.2	Bone pain 6 %
mTOR inhibitors						
TAMRAD (Bachelot et al. 2012)	ER/PgR-positive HER2-negative MBC	Aromatase inhibitor-resistant	111	Tamoxifen + everolimus	Clinical benefit rate = 61 %	Stomatitis 11 vs. 0 % Pain 9 vs. 18 % Infection 7 vs. 5 % Anorexia 7 vs. 4 %
				Tamoxifen	Clinical benefit rate = 42 %	
BOLER0-2/phase III (Baselga et al. 2012)	ER/PgR-positive advanced breast cancer	Aromatase inhibitor-resistant	724	Exemestane + everolimus	6.9	Stomatitis 8 vs. 1 % Anemia 6 vs. <1 % Dyspnea 4 vs. 1 % Hyperglycemia 4 vs. 1 % Fatigue 4 vs. 1 % Pneumonitis 3 vs. 0 %
				Exemestane + placebo	2.8	

*AE* adverse event, *CR* complete response, *EGFR* epidermal growth factor receptor, *ER* estrogen receptor, *HER2* human epidermal growth factor receptor 2, *MBC* metastatic breast cancer, *mTOR* mammalian target of rapamycin, *PFS* progression-free survival, *PgR* progesterone receptor, *PR* partial response, *SD* stable disease

^a^Study discontinued due to slow enrollment (94 out of 174 patients required)

^b^Stratum 1 of the trial included females with newly diagnosed metastatic disease or relapsed ≥1 year after stopping adjuvant tamoxifen

^c^Patients with centrally confirmed hormone receptor-positive, HER2-negative cancer (952 patients) had no improvement in PFS

One limitation of our trial is the relatively small sample size; however, this included 20 (71 %) HER2-negative patients, two of whom remained on treatment without progressing at 16 weeks. This observation is of interest because letrozole combined with lapatinib, a reversible EGFR and HER2 TKI, in the first-line setting failed to improve PFS in patients with ER-positive, HER2-negative tumors (Johnston et al. 2009; Schwartzberg et al. 2010). Importantly, ER-positive HER2-negative MBC patients with low ER expression demonstrated a statistically significant improvement in PFS when treated with letrozole plus lapatinib (Finn et al. 2009). Targeting HER2 with the monoclonal antibody trastuzumab and aromatase inhibitors such as anastrozole has also been explored, and the mammalian target of rapamycin (mTOR) inhibitor everolimus in combination with endocrine therapy represents another promising therapeutic strategy for ER-positive MBC (Table 5) (Huober et al. 2012; Marcom et al. 2007; Kaufman et al. 2009; Bachelot et al. 2012; Baselga et al. 2012). Understanding tumor biology and the compensatory mechanisms that are responsible for endocrine resistance will drive the choice of combination agent to provide optimal, synergistic blockade of relevant signaling pathways.

Across all doses of afatinib, the most frequently observed AEs were diarrhea, skin-related AEs, mucosal inflammation and asthenia. Overall, the side effect profile reported here was in accordance with the side effects observed across the phase I dose-finding afatinib monotherapy studies in patients with a variety of solid tumors, with GI and skin-related AEs most frequently reported (Yap et al. 2010; Eskens et al. 2008). These are known to be the most prominent AEs associated with EGFR blockade and are manageable (Mok et al. 2009; Mitsudomi et al. 2010; Frankel and Palmieri 2010; Rosell et al. 2012). Data from phase I monotherapy studies indicated an afatinib dose of 50 mg once daily to be the recommended dose for afatinib development in subsequent phase II studies (Yap et al. 2010; Agus et al. 2006; Eskens et al. 2008; Lewis et al. 2006). Therefore, patients who initially entered this phase II trial received a starting dose of afatinib 50 mg daily, with the option for dose reduction following the occurrence of specific grade 3 AEs. A notable proportion of patients initially treated with afatinib 50 mg in combination with letrozole experienced skin-associated AEs and diarrhea, requiring a dose reduction to 40 mg daily. The occurrence of significant skin-associated AEs and diarrhea within the 40 mg dose cohort also led to a subsequent dose reduction to 30 mg daily. The combination of afatinib/letrozole at a dose of 30/2.5 mg resulted in fewer patients discontinuing treatment due to AEs than patients who received 50 or 40 mg afatinib doses. Overall, the combination of afatinib and letrozole appeared to result in skin-related AEs occurring at greater intensity, when compared with afatinib monotherapy. It is worth noting that at the time of this study there was little known about the toxicity profile of TKIs and their management.

Co-administration of afatinib with letrozole resulted in afatinib pharmacokinetic parameters that were comparable to those observed during afatinib monotherapy (Yap et al. 2010; Eskens et al. 2008). Letrozole plasma concentrations were also comparable with or without afatinib administration and were within the ranges previously reported in the literature (Pfister et al. 2001; Dowsett 1999), indicating that there was no effect of afatinib on letrozole exposure. Therefore, this study is a preliminary indication that afatinib has no influence on the pharmacokinetics of letrozole and vice versa. However, the pharmacokinetic analysis was limited by the small number of patient samples and, as such, firm conclusions could not be drawn.

Despite the significant achievements of endocrine therapy in patients with breast cancer, only 50 % of all ER-positive tumors are initially responsive to anti-estrogens (Mouridsen et al. 2003; Osborne 1998). Furthermore, in the metastatic setting, tumors that are initially responsive always become resistant to endocrine treatment. Therefore, an agent that might suppress or delay the onset of endocrine resistance by disrupting multiple pathways at once and restoring hormone sensitivity may spare patients from treatment with aggressive chemotherapy. The phase II exploratory observations presented here show that the once-daily afatinib 30 mg/letrozole 2.5 mg combination was able to induce disease stabilization in 54 % of hormone-refractory MBC patients previously progressing on letrozole. This combination may warrant further evaluation, particularly in patients with ER-positive HER2-negative MBC. An ongoing study (NCT02115048) is assessing the efficacy of afatinib plus letrozole versus letrozole alone in first-line advanced ER-positive, HER2-negative postmenopausal breast cancer with low ER expression.

## Conclusions

In this small exploratory study, combination therapy with afatinib and letrozole led to disease stabilization in 54 % of patients with advanced hormone-refractory MBC who had progressed following letrozole treatment. These findings warrant further evaluation to determine the potential for overcoming the resistance associated with hormone therapy via ErbB Family blockade with afatinib.
